# Image quality of automatic coronary CT angiography reconstruction for patients with HR ≥ 75 bpm using an AI-assisted 16-cm z-coverage CT scanner

**DOI:** 10.1186/s12880-021-00559-7

**Published:** 2021-02-11

**Authors:** Cheng Yan, Guofeng Zhou, Xue Yang, Xiuliang Lu, Mengsu Zeng, Min Ji

**Affiliations:** 1 Department of Radiology, Zhongshan Hospital, Fudan University, Shanghai, China; 2 Shanghai Institute of Medical Imaging, Shanghai, China; 3 Department of Medical Imaging, Shanghai Medical College, Fudan University, Shanghai, China; 4 Shanghai United Imaging Healthcare Co., Ltd, Shanghai, China

**Keywords:** Computed tomography angiography, CT protocol, Artificial intelligence, Artifacts, Tomography, X-ray computed

## Abstract

**Background:**

Coronary CT angiography (CCTA) is a complicated CT exam in comparison to other CT protocols. Exam success highly depends on image assessment of experienced radiologist and the procedure is often time-consuming. This study aims to evaluate feasibility of automatic CCTA reconstruction in 0.25 s rotation time, 16 cm coverage CT scanner with best phase selection and AI-assisted motion correction.

**Methods:**

CCTA exams of 90 patients with heart rates higher than 75 bpm were included in this study. Two image series were reconstructed—one at automatically selected phase and another with additional motion correction. All reconstructions were performed without manual interaction of radiologist. A four-point Likert scale rating system was used to evaluate the image quality of coronary artery segment by two experienced radiologists, according to the 18-segment model. Analysis was done on per-segment basis.

**Results:**

Total 1194 out of the 1620 segments were identified for quality evaluation in 90 patients. After automatic best phase selection, 1172 segments (98.3%) were rated as having diagnostic image quality (scores 2–4) and the average score is 3.64 ± 0.55. When motion corrections were applied, diagnostic segment number increases to 1192 (99.8%) and the average score is 3.85 ± 0.37.

**Conclusions:**

With the help of 0.25 s rotation speed, 16-cm z-coverage and AI-assisted motion correction algorithm, CCTA exam reconstruction could be performed with minimum radiologist involvement and still meet image quality requirement.

## Background

With improvements in both hardware and algorithm, coronary computed tomography angiography (CCTA) has become a routine and noninvasive imaging method for coronary artery disease. There are several commercial cardiac-capable CT scanners with different engineering implementations. Single source CT (SSCT) with full field of view (FOV) is considered as most efficient and promising architecture to meet cardiac scan requirement [1]. For SSCT, single-beat scan has lower dose and do not require stable heart beat rate in comparison to multi-segment scan [2, 3]. But motion correction algorithm is required to further improve temporal resolution and reduce motion artifacts. This makes CCTA exam one of the most complicated CT protocols. In general, for 16-cm-coverage wide detector scanner, single-beat cardiac scan often consists of two steps—phase selection and motion correction. After data acquisition, radiologist will select a best reconstruction phase with lowest motion artifacts. Even with best phase option in most commercial CTs, radiologist often need to visually check the image quality and adjust reconstruction phase. After this subjective and time-consuming procedure, radiologist will then determine whether further motion correction need to be applied [4].

Recently a newly-developed 320-row scanner was introduced with 0.25 s rotation time (temporal resolution 125 ms). At the same time, a new generation AI-based motion correction was developed with improved correction quality and computing efficiency. It will be applied if there is still some motion artifact left. With all these advantages, we hypothesized that complex of CCTA scan workflow can be simplified.

In our study, we investigated the feasibility of one-stop CCTA exam without manual image evaluation during reconstruction using 320-row CT with 16-cm z-axis coverage and 0.25 s rotation time. For this purpose, we assessed the image quality of CCTA in challenging case (high heart rate) after auto-phase selection and motion correction in patients at high heart rate and analyzed the success rate based on image interpretability.

## Methods

### ePhase

**Fig. 1:**
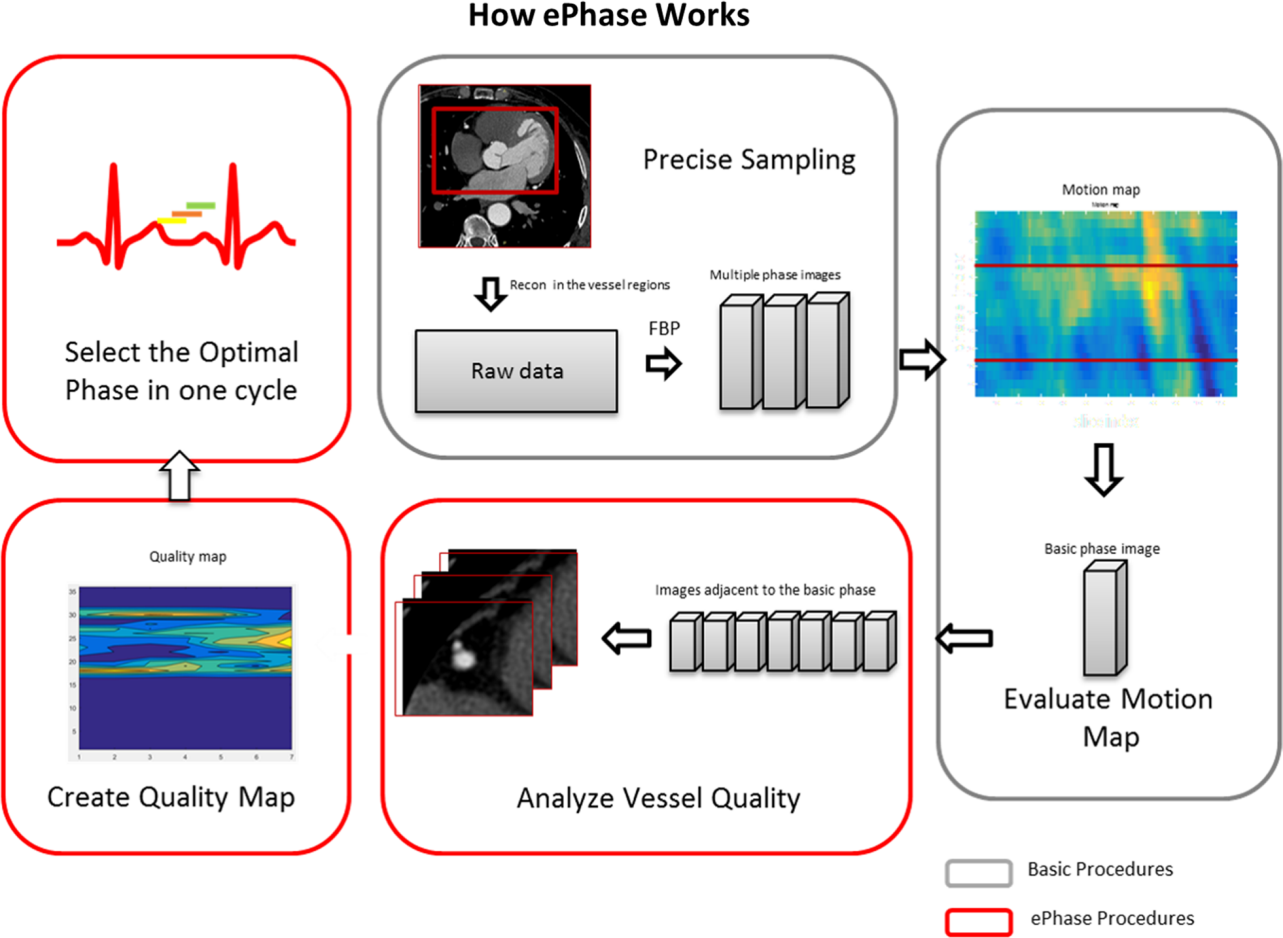
Workflow of ePhase

uCT 960+ is equipped with recently-developed automatic best phase selection method—ePhase. ePhase is a method based on coronary quality evaluation, besides measuring the image differences between phases [5]. Figure 1 is the flowchart of the ePhase algorithm. The gray boxes depict the ordinary process of the best phase selection. The red boxes show ePhase process. Like the mean absolute difference (MAD) algorithm [6], ePhase begins with a multi-phase reconstruction and a global criterion for the best phase selection. Precise sampling is done with a rapid multi-phase reconstruction step. It is performed focusing on the coronary artery using a small FOV and a small matrix size. The second step is the evaluation of motion map. The MAD is applied to obtain a motion map. The phase with the minimum value in that map is taken as the basic phase. The optimal phase range is determined by shifting near the basic phase. Although the determined phase of MAD does not necessarily coincide with the motion of the coronary, it can be used to limit the stable range of the cardiac cycles. Using the images of these phases, ePhase performs an automated extraction of the coronary for each image, and the quality is evaluated by calculating its circularity and sharpness. A good quality reconstruction of the coronary is achieved when the boundaries of the coronary are clear [7]. It was found that the coronary depicted with the lowest amount of motion will have a high regularity and boundary strength. This is reflected in its high-quality scores. Each image is evaluated and finally, a quality map can be obtained. The phase with the maximum score is considered the optimal phase. ePhase can provide equivalent image quality compared with experienced radiologist.

### CardioCapture

**Fig. 2:**
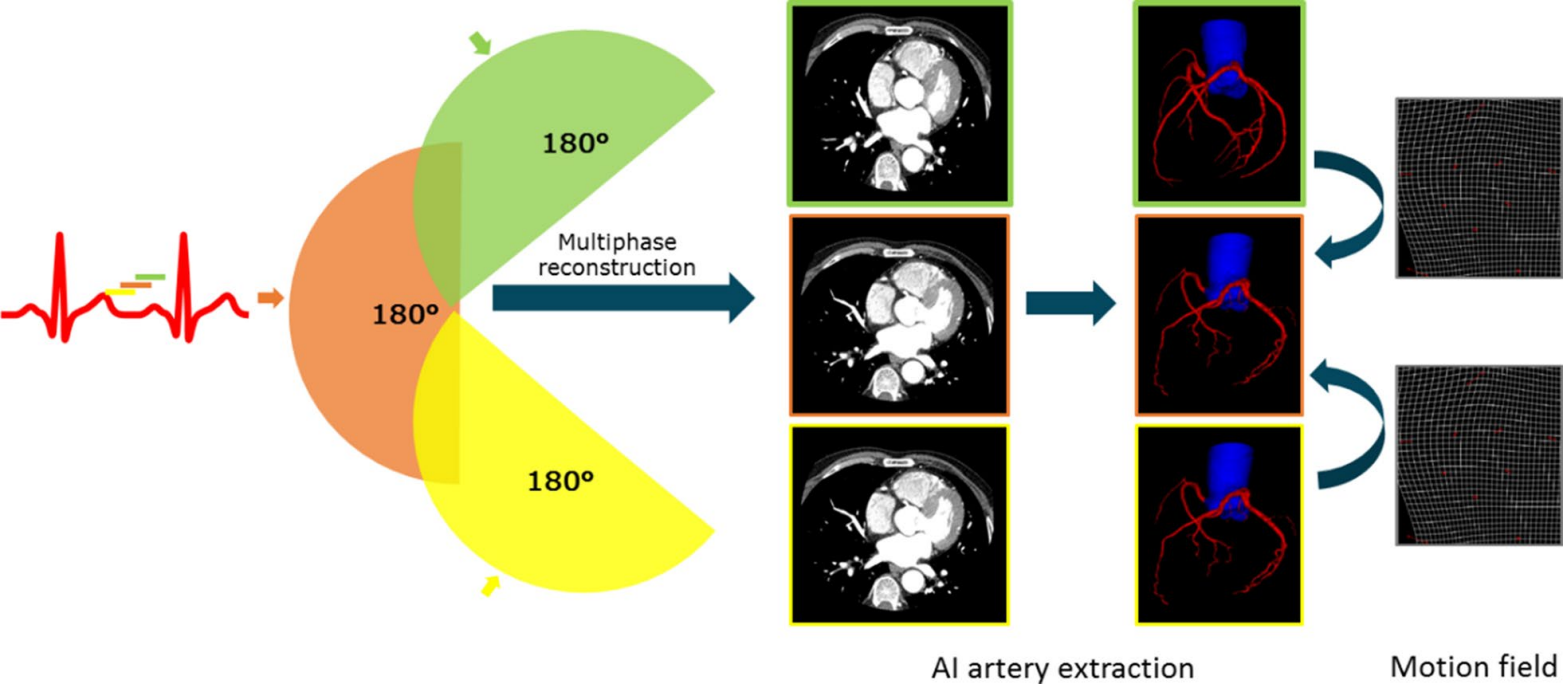
Workflow of CardioCapture

To further suppress the motion artifact, uCT 960+ includes a novel AI-assisted motion correction option—CardioCapture. Figure 2 is the flowchart of the motion correction algorithm—CardioCapture. Centerline based motion tracking/estimation is one of the main approaches in cardiac motion artifacts reduction. Centerline positions of coronary arteries are used to approximate the motion fields from one sampled phase to the selected phase. CardioCapture mainly focused on the motion correction around coronary arteries. Thus, centerline extraction of coronary arteries is one of the key procedures in CardioCapture algorithm. Benefiting from the performance and accuracy, deep learning with convolution neural networks (CNN) outperforms many traditional algorithms in image processing since 2016. In CardioCapture, a V-Net with dilated convolution [8, 9] was adopted for coronary artery segmentation. After that, a further optimization procedure was used to extract a smoothed centerline from the segmented coronary artery mask. After the centerline extraction step, a multi-level motion vector field generation scheme was adopted in the proposed solution and different scale of motion can be seized.

In principle, combining ePhase and CardioCapture, CCTA exam can be performed automatically, which is expected to improve exam efficiency and accuracy.

### Study population

This prospective study was approved by the Ethics Committee of Shanghai Zhongshan Hospital (Fudan University, Shanghai, China), and written informed consent was obtained from all participants before the commencement of the study. From May 2020 to August 2020, 90 consecutive patients with heart rates higher than 75 bpm during the CT scan was included in this study. Exclusion criteria included past history of allergy to the iodinated contrast agent, heart rate variability more than 20 bpm or arrhythmias, pregnancy, pacemaker and previous stent and/or bypass graft surgery. All exams were carried out in accordance with relevant guidelines and regulations.

### Scanning protocols

All examinations were performed on the newly introduced 320-row detector CT scanner (uCT 960+, United-imaging Healthcare) with prospectively electrocardiography triggering in a single heartbeat. Scanning parameters were as follows: z-coverage was 12 cm, 14 cm, or 16 cm depending on the patient’s heart size; reconstruction matrix size was 512 × 512 pixels; voxel size was 0.5 mm; gantry rotation time was 0.25 s; tube voltage was selected by AutokV option for 100 kV or 120 kV; tube current was determined by dose modulation (DOM) technique. Medium body was used with a scan field view of 420 mm to minimize the radiation dose. The display field-of view (DFOV) for the image reconstruction was 200 mm with an image slice thickness and increment of 0.5 mm. Data acquisition was triggered using a bolus-tracking technique, which started 6.0 s after the attenuation value in the descending aorta became higher than 110 Hounsfield units. Data acquisition covering 30–55% of the R-R interval was carried out. Non-ionic contrast media (iopamidol, 370 mg iodine/ml, Bayer) was injected at a rate of 4–5 ml/s via the median cubital vein, at a dose of 0.7 ml/kg of body weight and followed by 20 to 30 mL of saline solution. The dose and flow rate of the contrast agent were determined by the body mass index and vein condition. All selected patients were treated with oral metoprolol 1–1.5 h before the examination and sublingual nitroglycerine just before scanning.

### Image reconstructions

All images were reconstructed using the algorithm with commercial hybrid iterative reconstruction (KARL3D, Unite-imaging Healthcare) at level 3 for reducing image noise. The cardiac phase was selected automatically from the systolic phases by ePhase function and then additionally reconstructed with CardioCapture algorithm. Image quality of two image series after ePhase and CardioCapture were evaluated for further analysis.

### The radiation dose of CCTA

The effective radiation dosage of CCTA was calculated in millisieverts using a modified CT dose index volume specific for the CT scanner. The estimated effective dosage was calculated as the dose-length product times a conversion factor for the chest k = 0.014 (mSv × [mGy × cm] − 1) in the adult [10].

### Image evaluation

Two independent radiologists (with 8 and 11 years of experience in CCTA diagnosis) who were blinded to the reconstruction algorithms assessed the image quality of coronary segments. Image series after ePhase and CardioCapture were assessed in a random order with an interval of 2 weeks to reduce reader bias. Coronary artery segments with diameter of 1.5 mm or more were evaluated according to the 18-segment model, which is modified by the Society of Cardiovascular Computed Tomography [11]. Accordingly, a 4-point grading scale system was used to evaluate the image quality [4, 12]. Score 1 denotes non-diagnostic image quality (severe artifacts with inadequate delineation between the lumen and the surrounding tissue); 2 denotes adequate image quality (noticeably blurred vessel, but acceptable for diagnosis); 3 denotes good image quality (blurring of vessel margin and minor artifacts, fully evaluable); and 4 denotes excellent image quality (with the absence of artifacts). Coronary segment was considered non-diagnostic when the score was 1 and diagnostic from 2 to 4. For final statistical analysis, a consensus was reached after negotiation between the two radiologists, when there was difference in scoring.

### Statistical analysis

All statistical analyses were performed by SPSS software (version 22.0; SPSS, Chicago, Ill), Quantitative variables are presented as means ± standard deviations. The Kolmogorov–Smirnov test was used to test whether data were normally distributed. McNemar’s test was used to analyze statistical significance of the differences between paired proportions. Inter-observer agreement of subjective image quality score between the two radiologist readers was evaluated with the Cohen k test by using the following interpretation: *k* values of less than 0.20 were indicative of poor agreement; 0.21–0.40, fair agreement; 0.41–0.60, moderate agreement; 0.61–0.80, good agreement; and 0.81–1.00, excellent agreement.

## Results

### Study population

In total, 90 consecutive clinical coronary CT angiography examinations with heart rate > 75 bpm were evaluated. Table 1 describes the clinical characteristics of the 90 patients in this study. The mean patient age was 61.5 years ± 8.4; 44 of the 90 patients (48.9%) were men. The mean body mass index was 24.0 ± 2.3 kg/m^2^. The heart rate during the CT acquisition was 86.7 ± 6.7 bpm. Average effective dose (ED) was 2.2 ± 0.1 mSv. 88 out of 90 patients (97.8%) were scanned at 100kVp and 2 at 120 kVp (2.2%).

**Table 1:** Patient characteristics

n = 90	
Mean age	61.5 ± 8.4
Male	44
Female	46
BMI (kg/m^2^)	24.0 ± 2.3
Heart rate (bpm)	86.2 ± 6.7
CTDIvol (mGy)	10.0 ± 0.4
ED (mSv)	2.2 ± 0.1

### Image quality statistics

A total 1194 out of the 1620 segments were identified for evaluation in 90 patients with 426 (26%) segments being excluded (diameters < 1.5 mm). After ePhase reconstruction, among the 1194 segments, 1172 segments (98.3%) were rated as having diagnostic image quality (scores 2–4) and the average score is 3.64 ± 0.55 (Table 2). When CardioCapture were applied, diagnostic segments number increase to 1192 (99.8%) and the average score is 3.85 ± 0.37. The weighted kappa value for agreement between the two independent readers was 0.74 and 0.84 for ePhase and CardioCapture, respectively. In four major vessels (RCA, LM, LAD, LCX), CardioCapture could averagely improve IQ score by ~ 0.2 and ~ 0.9 for segments below 4 after ePhase (see details in Tables 3, 4).

**Table 2:** Segment number for each image quality scores after ePhase and CardioCapture (CC) (total 1194 segments)

Score	1	2	3	4
Segments after ePhase	20 (1.7%)	58 (4.9%)	250 (20.9%)	866 (72.5%)
Segments after CC	2 (0.2%)	6 (0.5%)	152 (12.7%)	1034 (86.6%)
*p*	< 0.001	< 0.001	< 0.001	< 0.001

**Table 3:** Score statistics of four major vessels after ePhase and CardioCapture(CC)

	RCA	LM	LAD	LCX
Mean score after ePhase	3.69 ± 0.65	3.89 ± 0.31	3.48 ± 0.87	3.55 ± 0.74
Mean score after CC	3.88 ± 0.34	4 ± 0	3.77 ± 0.72	3.84 ± 0.37
Mean difference	0.19	0.11	0.29	0.19
*p*	0.002	0.02	< 0.001	< 0.001

**Table 4:** Score statistics of four major vessels (scores below 4 after ePhase) after ePhase and CardioCapture(CC)

	RCA	LM	LAD	LCX
Mean score after ePhase	2.71 ± 0.67	3 ± 0	2.54 ± 0.86	2.65 ± 0.66
Mean score after CC	3.56 ± 0.56	4 ± 0	3.36 ± 1.01	3.51 ± 0.50
Mean difference	0.85	1.00	0.91	0.86
*p*	< 0.001	0	< 0.001	< 0.001

## Discussion

**Fig. 3:**
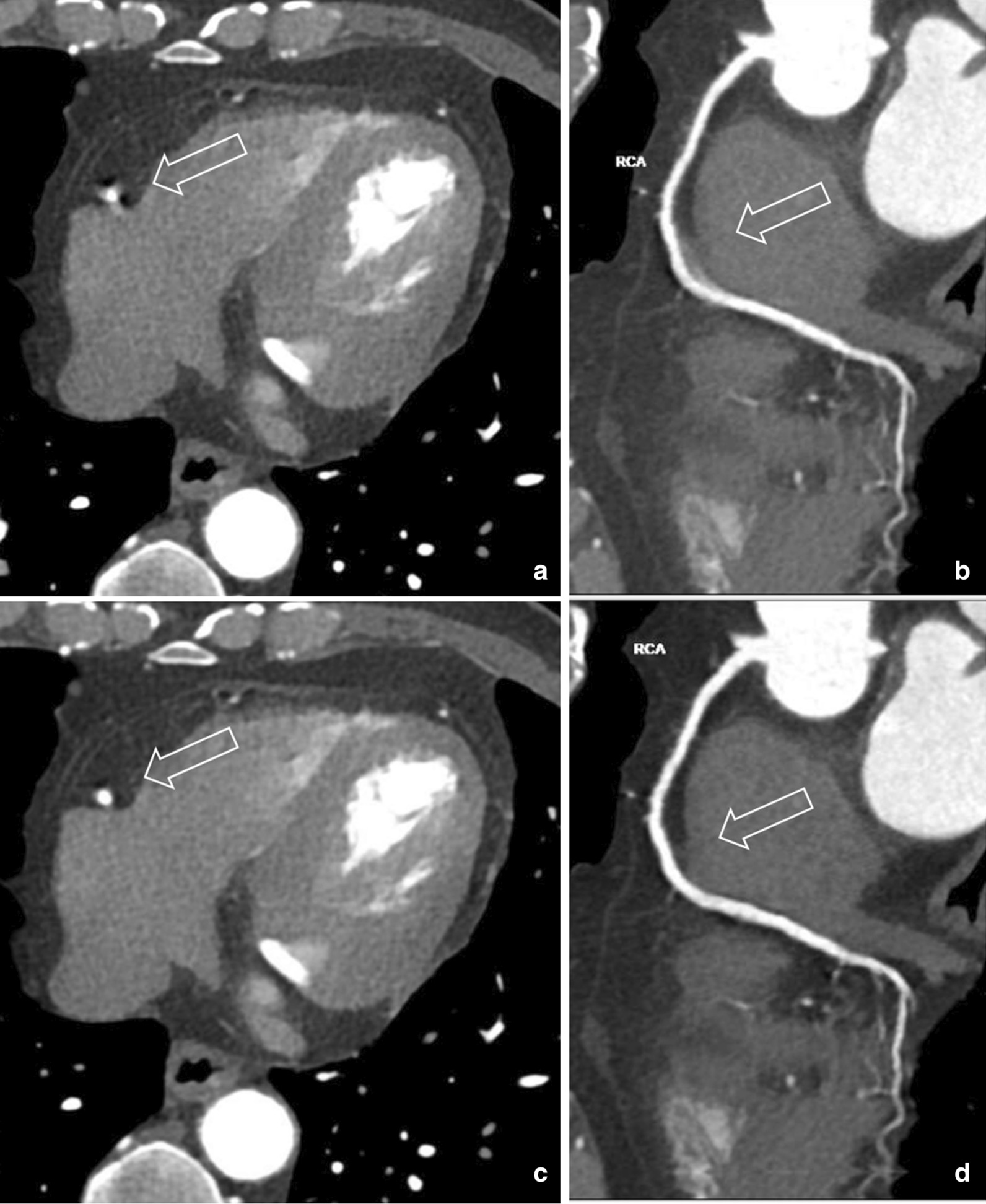
65-year-old man with heart rate 77 bpm. **a**, **b** After ePhase, mid right coronary artery (m-RCA) shows motion artifact, blurred edge. Curved planar reformation (CPR) image also blurred. Score rated at 2. **c**, **d** After CardioCapture, LAD motion artifact disappears, small vessel on CPR image is clear and sharp. Score rated at 4

**Fig. 4:**
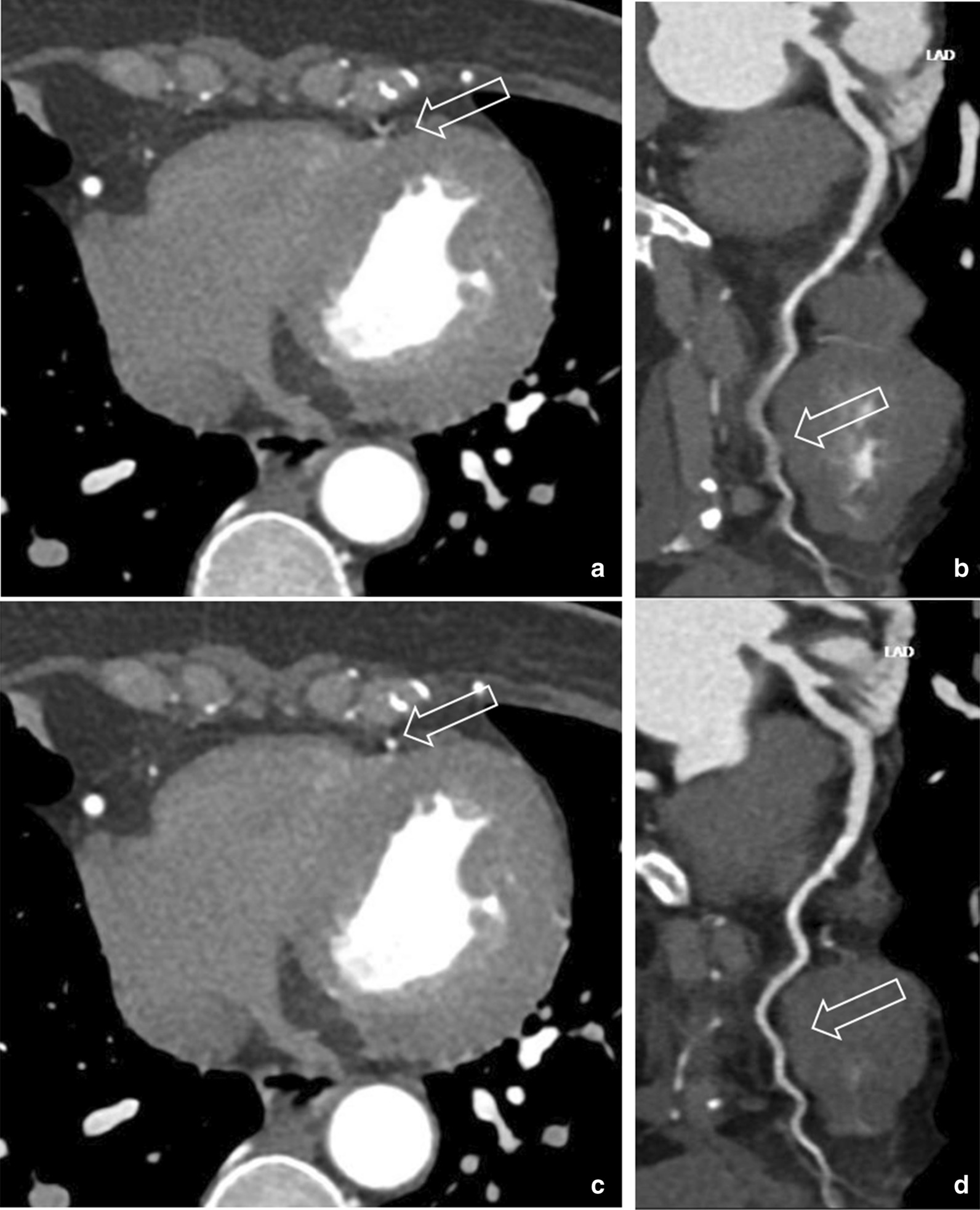
57-year-old woman with heart rate 86 bpm. **a**, **b** After ePhase: right coronary artery is clear, distal left anterior descending (d-LAD) coronary artery shows motion artifact, vessel boundary blurred. Score rated at 1 and non-diagnostic. **c**, **d** After CardioCapture: d-LAD motion artifact disappears, small vessel on CPR image is clear and sharp. Score rated at 3

Our preliminary study demonstrated that combining ePhase and CardioCapture, IQ of all patients with increasing heart rate in current study could meet diagnosis requirements without radiologist involvement. For patient with segments below score 4, CardioCapture could averagely increase rating by ~ 0.9. This demonstrated that CardioCapture can effectively remove most motion artifacts. Figures 3 and 4 show two example patient images after ePhase and CardioCapture. Although we only included the patients with high heart rate, but normally low heart rate patient was found less challenging in CCTA. In this sense, our proposed one-stop CCTA exam should perform better in such cases and then be feasible in most clinical situations.

In routine CCTA exam, when radiologist tries to find the optimal reconstruction phase, first he/she will choose one slice image which has apparent motion artifact at default or auto-selected phase reconstruction. Cardiac CT scanner normally provides ‘phase preview’ function which reconstructs several neighboring phases at selected slice position. Then radiologist selects the least motion artifact phase within the preview images and reconstructs the whole volume images for final verification. This procedure may be repeated for several times and sometimes inefficient because heart motion is complicated. Different segments or vessels does not move synchronously. Thus this ‘optimal’ phase is still subjective and limited. In contrast, computer could be more ‘objective’ and determine the image quality on whole artery vessel base efficiently. Furthermore, we also found that in some cases radiologist selected phase images will give even lower image quality after CardioCapture than automatic ePhase images. However, this topic is out of current study. It’s also worth mentioning that no image quality deterioration has been found in all cases. In this sense, with increasing computing power and more efficient AI algorithm, Motion correction could be routinely applied in all CCTA exams and dramatic changes in CCTA exam workflow with improving hardware and algorithm, especially AI technique, are expected.

In comparison with several previous studies [4, 13–16] on whole-heart coverage CT scanner, our overall segment scores are a little bit higher which could be due to faster rotation speed or radiologist assessment bias. But in general, the average score increases after motion correction is similar. In some works, phase selection before motion correction was done by an experienced radiologist [4] or details for phase selection were not provided [13, 15, 16]. In one study, the best cardiac phase and motion correction were both applied but this study focused on low kVp image quality and study population was relatively smaller (n = 30) [14]. To our knowledge, currently radiologist image assessment during CCTA exam is still required in general clinical practice.

There were some limitations to this study. First, sample size of patients in our study was not quite large and single center-based, which may affect image quality scores. But considering we focused on high heart rate cohort population, current case number was comparable to several previous studies. Second, patients with irregular heart rates were excluded. Irregular heart rate is still challenging for phase selection in both acquisition and reconstruction steps. Further investigation will be required to demonstrate the feasibility of automatic exam in such case. In current clinical practice, radiologist could pre-determine whether automatic exam is doable by checking the ECG signals. Third, our study was focused on qualitative image quality alone. Larger population study and diagnostic accuracy study such as ICA comparison will be carried out in the future. Fourth, this study is limited to segments with a diameter > 1.5 mm. Though this exclusion is common practice in CCTA, small vessel disease diagnosis could be influenced by motion artifact.

## Conclusions

In conclusion, with the help of fast rotation speed (0.25 s/rot), 16-cm z-coverage and AI-based motion correction algorithm, CCTA exam reconstruction can be performed with minimum radiologist involvement and still meet image quality requirement. We can expect in near future, this challenge exam in CT will be fully automatic and benefit patient in more clinical situations.

## Data Availability

The datasets used or analyzed during the current study are available from the corresponding author on reasonable request.

## Abbreviations

CCTA: Coronary computed tomography angiography
bpm: Beats per minute
SSCT: Single source CT
RCA: Right coronary artery
LCX: Left circumflex artery
LAD: Left anterior descending artery
LM: Left main artery
CNN: Convolution neural networks
AI: Artificial intelligence
MAD: Mean absolute difference
ED: Effective dose
